# Evaluation of Pathogenic Variants Associated With Monogenic Disorders of Dyslipidemia in Patients With Well Characterised MASLD

**DOI:** 10.1111/liv.70486

**Published:** 2026-01-09

**Authors:** Tae‐Hwi Schwantes‐An, Marco A. Abreu, Brent A. Neuschwander‐Tetri, Jian Wang, Xiuqing Guo, Jingyi Tan, Robert A. Hegele, Nicholas O. Davidson, Luca Lotta, Niek Verweij, Katherine P. Yates, Jerome I. Rotter, Naga Chalasani

**Affiliations:** ^1^ Department of Medical and Molecular Genetics Indiana University School of Medicine Indianapolis Indiana USA; ^2^ Division of Gastroenterology and Hepatology Saint Louis University St Louis Missouri USA; ^3^ Robarts Research Institute, Schulich School of Medicine & Dentistry Western University, 1151 Richmond St London Ontario Canada; ^4^ Institute for Translational Genomics and Population Sciences, Los Angeles Biomedical Research Institute at Harbor‐UCLA Medical Center Torrance California USA; ^5^ Department of Biochemistry Schulich School of Medicine and Dentistry, Western University London Ontario Canada; ^6^ Department of Medicine, Schulich School of Medicine and Dentistry Western University London Ontario Canada; ^7^ Division of Gastroenterology, Department of Medicine Washington University School of Medicine Saint Louis Missouri USA; ^8^ Regeneron Genetics Center, Regeneron Pharmaceuticals Tarrytown New York USA; ^9^ Department of Epidemiology and Biostatistics Johns Hopkins University Baltimore Maryland USA; ^10^ Division of Gastroenterology and Hepatology, Department of Medicine Indiana University School of Medicine Indianapolis Indiana USA

**Keywords:** *APOB*, hereditary dyslipidemia, histological progression, *LDLR*, MASLD

## Abstract

**Background and Aims:**

Dyslipidemia is common in patients with MASLD, but the frequency and significance of inherited disorders of dyslipidemia are unclear. We investigated the prevalence and significance of pathogenic variants associated with selected monogenic disorders of dyslipidemia in 3358 patients with well‐characterised MASLD.

**Approach:**

We identified clinically relevant variants in *APOB*, *MTTP*, *PCSK9*, *ANGPTL3*, *LDLR* and *LDLRAP1* genes which can cause hypobetalipoproteinemia (HBL) and familial hypercholesterolemia (FH). Using ClinVar annotations as initial variant selection, we identified 2027 variants in those 6 genes which are reported as ‘pathogenic’ or ‘likely pathogenic’ (P/LP). We first assessed for the presence of P/LP variants in the study cohort and then investigated the effect of carrying P/LP variants on liver histology, by comparing ~4 matched controls for each *APOB* and *LDLR* carrier. As interpretative analyses, we also looked at the difference between liver enzymes, lipid measures and outcomes between the carriers and matched controls.

**Results:**

Twenty‐two variants among these 2027 P/LP variants were present in 24 out of 3358 patients (12 *ApoB*, 10 *LDLR*, 1 *ANGPTL3* and 1 *MTTP* variant carriers). Compared to controls, *APOB* carriers had higher steatosis grade (2.4 vs. 1.7, *p*‐value 0.0028), higher NAFLD activity score (NAS) (4.9 vs. 3.8, *p*‐value 0.04), and numerically higher but statistically not significant fibrosis stage (1.2 vs. 1.1, *p*‐value 0.75) and ALT (87.4 vs. 58.1 U/L, *p*‐value 0.06). Their LDL‐c (51 vs. 147.8 mg/dL, *p*‐value 6.1E‐09) and triglycerides (91.5 vs. 160.6 mg/dL, *p*‐value 2.8E‐03) were significantly lower. Compared to controls, *LDLR* carriers had numerically higher steatosis grade, NAS, fibrosis stage and LDL‐c levels, but these were not statistically different.

**Conclusions:**

Monogenic disorders of dyslipidemia are rarely present in patients with MASLD and are sometimes associated with worse liver histology. Testing for these conditions may be considered on a case‐by‐case basis.

## Key Points

Genetic variants that cause dysregulation of lipids are not more prevalent among the patients with MASLD, liver diseases driven by metabolic dysfunctions such as lipid accumulation. Some of the variants (e.g., *APOB*) do increase risk for worse liver histology. Although these variants may explain some of the etiologies in MASLD, additional studies are required to elucidate their role in MASLD.

## 1 Introduction

Dyslipidemia is common in patients with metabolic dysfunction associated steatotic liver disease (MASLD), characterised by elevated apolipoprotein (apo) B‐containing lipoproteins that are associated with increased risk of atherosclerotic cardiovascular disease (ASCVD) [1]. The dyslipidemia in MASLD patients is considered to be the consequence of overproduction of hepatic apo B‐containing lipoproteins, primarily very‐low density lipoprotein (VLDL), which is remodeled to low‐density lipoprotein (LDL) in the circulation [2]. The best‐known human genetic dyslipidemia is familial hypercholesterolemia (FH), in which elevated LDL cholesterol and apo B result not from overproduction but rather defective catabolism due to loss‐of‐function variants in the *LDLR* gene encoding the LDL receptor, or less frequently in the *APOB, PCSK9* and *LDLRAP1* genes encoding apo B or proprotein convertase subtilisin kexin type 9 (*PCSK9*) and LDL receptor adaptor protein 1 (*LDLRAP1*), respectively [3]. FH is associated with premature ASCVD in both adults and children [4, 5] but typically not with MASLD. In contrast, patients with the mirror‐image phenotype, namely recessive abetalipoproteinemia or homozygous hypobetalipoproteinemia, due to pathogenic variants in the *MTTP* or *APOB* gene, respectively, characterised by failure to produce and secrete apo B‐containing lipoproteins, are protected from ASCVD but can sometimes display hepatic steatosis and eventually hepatic fibrosis [6, 7, 8]. Vilar‐Gomez et al. reported patients who carry a single copy of a rare variant in *APOB* that impairs structural integrity of lipoprotein particles causing heterozygous hypobetalipoproteinemia had increased aminotransferase (ALT) levels compared to those without such variants [4]. This is consistent with prior studies showing varying degrees of liver injury and fibrosis in hypobetalipoproteinemia [5, 6, 9], especially in the setting of insulin resistance [7]. Pathogenic variants in *ANGPTL3* encoding angiopoietin‐like protein 3 are also associated with reduced levels of apo B‐containing lipoproteins and protection from ASCVD, although hepatic involvement has not been consistently demonstrated [8].

Although pathogenic variants underlying extreme phenotypes of apo B‐containing lipoproteins are associated with ASCVD and hepatic phenotypes, the association of such variants with the spectrum of MASLD has not been comprehensively explored. Therefore, we evaluated the relationship of pathogenic or likely pathogenic variants in *APOB*, *MTTP*, *PCSK9*, *ANGPTL3*, *LDLR* and *LDLRAP1* genes [10, 11, 12, 13] and liver histology in patients with MASLD. In a biopsy‐proven cohort of patients with MASLD from the NASH‐CRN cohort, we evaluated the association between the clinically relevant variants in these genes with liver histology and plasma lipid levels.

## 2 Methods

### 2.1 Study Participants

We included both adult and paediatric patients with biopsy confirmed MASLD (metabolic dysfunction‐associated steatotic liver disease) from the NASH‐CRN (Nonalcoholic Steatohepatitis Clinical Research Network) who were prospectively recruited at multiple medical centers across the United States between 2004 and 2020. The diagnosis of MASLD was based on > 5% of hepatocytes containing macrosteatosis and exclusion of significant alcohol consumption (> 20 g/d for women, > 30 g/d for men) within 2 years of the initial biopsy. All liver biopsies were reviewed in a blinded fashion by the NASH‐CRN Pathology Committee and scored according to the NASH‐CRN Scoring System [14]. For this analysis, the key inclusion criteria were having available blood DNA with QC (quality control) passing WES (whole exome sequence) and targeted WGS (whole genome sequencing) along with available phenotype measurements. Additional details of the NASH‐CRN participants' demographic, alcohol consumption, medical history, lab tests, liver biopsy results, prescription information and study inclusion and exclusion criteria have been described [15].

### 2.2 Phenotype Data

All reported liver‐related laboratory values, diagnoses, body measurements and histology results were obtained at the time of enrolment. To adjust for the effect of statin therapy on serum total cholesterol levels, we imputed the amount of reduction proposed by Ruel et al. [16]. For those participants on any statin, we adjusted their LDL‐C (Low‐density lipoprotein cholesterol) by dividing LDL‐C values by 0.52 (on average, 48% reduction is expected on statin therapy). Fibrosis stage was assessed from 0 (no fibrosis) to 4 (cirrhosis). Steatosis was graded from 0 to 3, ballooning was graded from 0 to 2 and inflammation from 0 to 3; the sum of these three measures defined the NAFLD Activity Score (NAS). For the purposes of this analysis, a histologic diagnosis of definite steatohepatitis was grouped with borderline steatohepatitis and compared to not‐steatohepatitis. The additional prospectively recorded histologic features of lobular inflammation, portal inflammation, ballooning and iron staining were dichotomized into present versus not present.

### 2.3 Genotype Data

Whole Exome Sequencing (WES) data was generated, and primary analysis of variant calling was performed by the Regeneron Genetics Center (RGC) as published previously [17]. In short, WES plus targeted variant sequencing was conducted using Illumina v4 HiSeq2500 or NovaSeq instruments using an exome plus targeted set of variants capture kit. WES variants were filtered using RGC's Goldilocks pipeline [18]. The targeted variants (common variants) were cleaned using a previously published GWAS (genome‐wide association study) genotype data cleaning pipeline [19]. Both variants and samples are filtered for low genotyping rate (< 95%), both expected and unexpected relationships were checked, and HWE (Hardy–Weinberg Equilibrium) were checked to ensure the variants that deviated from HWE were removed. Cleaned genotype data was then imputed using TOPMed imputation server [20].

### 2.4 Curation of Clinically Relevant Variants

To identify clinically relevant variants which have been reported to be associated with dyslipidemia, we targeted 6 genes that were previously reported to have pathogenic variants for hypobetalipoproteinemia (HBL) and/or familial hypercholesterolemia (FH): *APOB* (Apolipoprotein B), *MTTP* (Microsomal Triglyceride Transfer Protein), *PCSK9* (Proprotein Convertase Subtilisin/Kexin Type 9), *ANGPTL3* (Angiopoietin Like 3), *LDLR* (Low Density Lipoprotein Receptor) and *LDLRAP1* (Low Density Lipoprotein Receptor Adaptor Protein 1). We used ClinVar [21] for initial variant screening to identify variants in the 6 genes that have been reported to be ‘*pathogenic*’ or ‘*likely pathogenic*’ (P/LP) for conditions that included ‘*hypobeta*’ or ‘*hypercholesterolemia*’ in its names. In total, there were 2027 clinically relevant variants which were used to identify carriers for analysis.

### 2.5 Identifying the Carriers of Clinically Relevant Variants Among NASH‐CRN Patients

To identify carriers of alternative alleles in the clinically relevant variants from the 6 genes, we first mapped which clinically relevant variants were sequenced in NASH‐CRN WES data. First, the clinically relevant variants' physical location, chromosome and starting/ending base position were overlapped to the QC passing list of variants from NASH‐CRN WES data. Second, for those overlapping positions, single nucleotide variants (SNVs) were required to have matching REF/OTHER alleles (or complementary bases to both REF/OTHER alleles) while InDels (short Insertions and Deletions) were required to have the corresponding allele changes (e.g., may have different length alleles but the changed bases are the same between clinically relevant variant and NASH‐CRN genotypes). Other variants such as larger insertions/deletions, microsatellites, or CNVs (copy number variants) were not mapped since our WES data was not used to call those variants. Following these steps, we identified 22 variants (16 SNVs and 6 InDels) in 4 genes (*APOB*, *ANGPTL3*, *LDLR*, *MTTP*) from the clinically relevant variants in NASH‐CRN WES data. For the 4 genes, NASH‐CRN individuals were marked as carrier if a participant carried the alternate (ALT) allele among the mapped variants in each gene. Those participants who did not carry any of the mapped variants for each gene were marked as non‐carrier. There were no carriers of *PCSK9* or *LDLRAP1*.

### 2.6 Matched Non‐Carriers/Controls

To interrogate the significance of having these clinically relevant variants, we identified a set of controls, the participants who did not carry the clinically relevant variants, from NASH‐CRN to compare to the carriers. We matched non‐carriers to carriers for age, sex and an unweighted three‐locus single nucleotide polymorphism (SNP) risk score which tallied the number of risk alleles in rs738409 (*PNPLA3*, G allele), rs72613567 (*HSD17B13*, T allele) and rs58542926 (*TM6SF2*, T allele) for a score ranging between 0 to 6. For comparisons, we matched approximately 4 non‐carriers to each carrier of *ApoB* or *LDLR*.

### 2.7 Statistical Analyses

All statistical analyses were conducted using R [22]. Wilcoxon rank sum test was used to compare the continuous variable between the carriers and matched non‐carriers. For the categorical variables, Fisher's Exact test was conducted. As primary outcomes of interest, we compared steatosis grade, fibrosis stage and NAS between *APOB* carriers versus *APOB* non‐carriers and *LDLR* carriers versus *LDLR* non‐carriers, leading to a total of 6 discovery tests. After multiple testing correction using Bonferroni's correction, the statistical significance threshold was 0.008 (0.05/6).

### 2.8 UK Biobank PDFF Versus Carriers Test

To further interpret our findings, we looked at the effects of carrying clinically relevant variants in *APOB* or *LDLR* in the UK Biobank cohort. We screened the individuals who had both MRI (magnetic resonance imaging) based PDFF (proton density fat fraction) data and WES data available. In total, there were 41 096 participants available for comparing PDFF and variant carrier status. PDFF was dichotomized at ≥ 5% versus < 5%. Carriers for *APOB* and *LDLR* were identified using the same set of variants used to identify carriers for the two genes in NASH‐CRN patients. Fisher's exact test was conducted to compare between carrier status and dichotomized PDFF phenotype.

## 3 Results

In total, there were 2027 clinically relevant variants identified in our search. Of the 2027 variants, 22 variants from 4 genes were identified among 3358 NASH‐CRN study participants who had available genetic data. Table S1 shows the 22 variants with their relative positions in each gene, Canonical SPDI (sequence, position, deletion, insertion based variant ID), HGVS nomenclature, ClinVar ID and ACMG classification. Upon screening for the carrier status for the 22 variants, there were a total of 24 NASH‐CRN participants who carried an ALT allele in the 22 variants. There were no multiple allele carriers. There were 12 carriers of *APOB* variants, 10 carriers of *LDLR* variants and one carrier each for *ANGPTL3* and *MTTP* variants. Table S2 provides detailed clinical and genetic information on the 24 carriers. Using the matching algorithm, we identified 45 matched controls for the 12 carriers of *APOB* and 37 matched controls for the 10 carriers of *LDLR* variants. Table 1 shows demographics, diagnoses and liver‐related laboratory values for overall, *APOB* carriers, *LDLR* carriers and their matched controls. Overall, there were no statistically significant (*p*‐value < 0.05) differences between the two carrier groups and their matched controls except for the lipid measurements. As expected, LDL cholesterol level was significantly lower in the carriers of variants in *APOB* compared to the non‐carriers (51 mg/dL vs. 148 mg/dL, respectively, *p*‐value = 6.08 × 10^−09^), consistent with the known biochemical phenotype of heterozygous hypobetalipoproteinemia along with low total cholesterol levels. Triglyceride levels were also significantly lower in both *APOB* and *LDLR* carriers group compared to respective matched controls groups. Adults and paediatrics stratified results are shown as Tables S3 and S4. Among the two carriers who did not have variants in *APOB* or *LDLR*, the *MTTP* variant carrier showed elevated triglyceride and cholesterol levels and BMI of 34 kg/m^2^ and the *ANGPTL3* variant carrier showed normal triglyceride and cholesterol but elevated BMI (32 kg/m^2^) and had type‐2 diabetes diagnosis (Table S2).

**TABLE 1:** Summary of *APOB* and *LDLR* variant carriers and matched controls.

Variable	Overall (*N* = 3358)	APOB carriers (*N* = 12)	APOB matched controls (*N* = 45)	APOB versus matched controls *p*‐value	LDLR carriers (*N* = 10)	LDLR matched controls (*N* = 37)	LDLR versus matched controls *p*‐value
Age at enrolment, mean (SD)	39.9 (19.8)	40.1 (15.9)	41.6 (14.9)	0.78	43.6 (20.3)	42.5 (19.3)	0.88
% Male (*N*/total)	47.5% (1595/3358)	16.7% (2/12)	11.1% (5/45)	0.63	40% (4/10)	43.2% (16/37)	1.00
% Self‐reporting White	76% (2553/3358)	83.3% (10/12)	66.7% (30/45)	0.32	80% (8/10)	73% (27/37)	1.00
% Self‐reporting Hispanic	28.6% (961/3358)	8.3% (1/12)	15.6% (7/45)	1.00	30% (3/10)	37.8% (14/37)	0.73
% Paediatric pt	28.4% (953/3358)	16.7% (2/12)	17.8% (8/45)	1.00	20% (2/10)	21.6% (8/37)	1.00
% on Statin	21.8% (730/3354)	0% (0/12)	35.6% (16/45)	0.01	20% (2/10)	24.3% (9/37)	1.00
BMI	33.7 (6.6)	33.4 (8.4)	35.7 (7.4)	0.40	35.4 (9.1)	33.6 (6.7)	0.57
% Diabetes	27.8% (932/3354)	16.7% (2/12)	44.4% (20/45)	0.10	30% (3/10)	40.5% (15/37)	0.72
% CAD	3.2% (108/3354)	0% (0/12)	6.7% (3/45)	1.00	0% (0/10)	0% (0/37)	1.00
% CVD	3.8% (127/3354)	0% (0/12)	6.7% (3/45)	1.00	0% (0/10)	0% (0/37)	1.00
% CKD	2.5% (85/3354)	0% (0/12)	4.4% (2/45)	1.00	10% (1/10)	8.1% (3/37)	1.00
ALT in U/L	79 (65)	87.4 (44.7)	57.5 (47.2)	0.06	87.6 (47.7)	77 (86)	0.63
AST in U/L	53.5 (38.7)	57.2 (23.3)	43.4 (29.9)	0.10	58.1 (29.3)	52.2 (44.1)	0.63
AlkPhos in U/L	124 (88)	110.7 (79)	120.2 (82.6)	0.72	118 (92.9)	120.8 (84.3)	0.94
Bilirubin in mg/dL	0.6 (0.4)	0.5 (0.2)	0.5 (0.2)	0.95	0.6 (0.3)	0.5 (0.3)	0.64
GGT in U/L	63.6 (76)	61.7 (31.5)	56.7 (33.2)	0.64	66.4 (46.6)	77.1 (97.1)	0.64
HDL in mg/dL	42.8 (11.9)	54 (17.8)	46.8 (16.8)	0.22	40.4 (8.5)	44.5 (12.9)	0.27
LDL in mg/dL	128.3 (56.8)	51.1 (25.3)	147.8 (77)	6.08E‐09	156.9 (93.3)	127 (42.4)	0.37
Total Cholesterol in mg/dL	202.7 (62.8)	124.6 (29.2)	226.7 (83.3)	2.28E‐08	221.9 (93.7)	205.3 (48.1)	0.62
Triglyceride (mg/dL)	167.8 (129.5)	91.5 (60.2)	160.6 (68.7)	2.77E‐03	122.9 (57.4)	209.5 (186)	0.02
Systolic BP in mmhg	128.1 (15.7)	123.8 (10.8)	126.2 (13.9)	0.53	130 (29)	125.3 (13.4)	0.63
Diastolic BP in mmhg	74 (11)	74.2 (6.8)	72.1 (11.1)	0.43	77.5 (12.9)	74.9 (9)	0.56
FNI% (SD)	0.5 (0.3)	0.5 (0.3)	0.4 (0.3)	0.45	0.6 (0.3)	0.4 (0.3)	0.25

*Note:* Summary of table of demographics, clinical diagnoses and liver lab values between *APOB* and *LDLR* variant carriers and their matched controls. Overall, we did not observe any statistically significant differences between the groups (*p* value < 0.05) except for % on Statin, LDL, Total Cholesterol and Triglyceride in *APOB* carriers versus matched controls and Triglyceride levels in *LDLR* carriers versus matched controls. *APOB* versus matched controls *p*‐value and *LDLR* versus matched controls *p*‐value columns show Wilcoxon rank sum *p*‐values for continuous variables and Fisher's exact *p*‐value for categorical variables.

Abbreviations: AlkPhos, alkaline phosphatase; ALT, alanine aminotransferase; AST, aspartate aminotransferase; BMI, body mass index; BP, blood pressure; CAD, coronary artery disease; CKD, chronic kidney disease; CVD, cardiovascular disease; FNI, fibrotic NASH index; GGT, gamma‐glutamyl transferase; SD, standard deviation.

For primary outcomes of interest, we compared steatosis grade, fibrosis stage, and NAS between the carriers and matched non‐carriers for *APOB* and *LDLR* genes. After Bonferroni correction, we observed higher steatosis grade among *APOB* carriers (*N* = 12) compared to the matched controls (*N* = 45, *p*‐value = 0.0028, Table 2). NAS was higher in *APOB* carriers (NAS = 4.9) compared to the matched controls (3.8), but it was not statistically significant post multiple‐testing correction (*p*‐value = 0.04). Fibrosis stage was not different between *APOB* carriers and matched non‐carriers (1.2 vs. 1.1, respectively, *p*‐value = 0.75). *LDLR* carriers and matched non‐carriers did not show differences in steatosis grade, fibrosis stage, nor NAS (Table 2).

**TABLE 2:** Association between *APOB* and *LDLR* carrier status and histology measures.

Variable	APOB carriers (*N* = 12)	APOB matched controls (*N* = 45)	APOB versus matched controls *p*‐value	LDLR carriers (*N* = 10)	LDLR matched controls (*N* = 37)	LDLR versus matched controls *p*‐value
Steatosis grade	2.4 (0.7)	1.6 (0.9)	2.77E‐03	2.3 (0.7)	1.8 (0.8)	0.06
Fibrosis stage	1.2 (1.4)	1.1 (1.3)	0.75	1.9 (1.5)	1.5 (1.3)	0.51
NAS	4.9 (1.4)	3.8 (1.7)	0.04	5 (1.9)	4.1 (1.8)	0.20
Lobular inflammation	66.7% (8/12)	28.9% (13/45)	0.02	50% (5/10)	40.5% (15/37)	0.72
Portal inflammation	91.7% (11/12)	84.4% (38/45)	1.00	90% (9/10)	94.6% (35/37)	0.52
Steatosis grade ≥ 2	91.7% (11/12)	48.9% (22/45)	0.01	90% (9/10)	54.1% (20/37)	0.06
Ballooning	58.3% (7/12)	53.3% (24/45)	1.00	60% (6/10)	45.9% (17/37)	0.49

*Note:* Associations between *APOB* and *LDLR* carrier status and liver histology lesions. Post multiple‐testing corrected *p*‐value significance threshold was 0.008 (for Steatosis, Fibrosis and NAS). Lobular and portal inflammation, dichotomized steatosis grade and ballooning were added to aid the interpretation of the significant associations between *APOB* carrier status and Steatosis and NAS. Steatosis grade, fibrosis stage and NAS were treated as continuous variables. Controls were matched for age, sex, and three‐SNP risk score.

As secondary, or exploratory analyses, we tested whether carrier status was associated with lobular inflammation, portal inflammation and ballooning (Table 2). Among the liver histology measures, only lobular inflammation was significantly different between carriers and non‐carriers of P/LP variants in *APOB*: 67% versus 29%, respectively (*p*‐value = 0.02). We also looked at mortality and liver related outcomes in the carriers and matched controls. Among *APOB* carriers and matched controls, the median years of follow up was 5.1 years (interquartile range 1.9 to 5.8 years) among the carriers and 4 years (1.1 to 8.0 years) among the matched controls. There were no deaths among the carriers while among the matched controls there were three liver related deaths. Among *LDLR* carriers and matched controls, the median year of follow up was 1.7 years (0.3 to 9.2 years) for the carriers and 3.3 years (1.2 to 7.2 years) for the matched controls with no deaths among the carriers and 1 liver related death among the matched controls.

To further characterise the association between steatosis grade and carrying clinically relevant P/LP variants in *APOB*, we conducted an analysis comparing individuals with high PDFF (Proton Density Fat Fraction, PDFF ≥ 5%) and low PDFF (< 5%) from the UK Biobank. In total, there were 6 carriers of the 10 variants in *APOB*, 41 carriers of 9 variants in *LDLR* and 41 096 individuals with a ≥ 5% PDFF value who did not carry any of the 22 screened variants. Carrying a P/LP variant in *APOB* showed a numerically higher risk for having higher PDFF (odds ratio (OR) = 2.6, 95% Confidence Interval (95% CI) = 0.5–12.8, *p*‐value = 0.36) while *LDLR* carriers showed a protective trend (0.4, 95% CI 0.2–1.1, *p*‐value = 0.08) for having a high PDFF value.

## 4 Discussion

The principal finding of this study in the NASH‐CRN cohort is a significant association between heterozygosity for a P/LP variant in *APOB* causing hypobetalipoproteinemia and increased steatosis grade: 2.4 versus 1.6 (*p*‐value 0.0028). Although not statistically significant, we found similar trends in the UK Biobank among individuals who carry the P/LP variants in *APOB* for increased risk of higher PDFF value (OR = 2.6) compared to those without the variants. Also, heterozygosity for P/LP variants in *APOB* was nominally associated with a higher NAFLD activity score (4.9 vs. 3.8, *p*‐value 0.04). Taken together, our findings suggest that pathogenic variants underlying hypobetalipoproteinemia are associated with worse liver histology among patients with MASLD. However, we did not observe any statistically significant associations with *LDLR* variants (all *p*‐values > 0.05) and histological characteristics.

These observations were made with matched non‐carrier controls, adjusting for age, sex and the contribution from the common genetic risks based on a three‐SNP genetic risk score for hepatic steatosis including *PNPLA3*, *TM6SF2* and *HSD17B13* [23]. These findings suggest that the genetic variants which lead to low LDL cholesterol and apo B‐containing lipoproteins may have a role in histological progression among patients diagnosed with MASLD independent of previously known common genetic variants.

Although we utilised a well characterised cohort of patients with MASLD, there are a few weaknesses in the current study. First, the number of carriers was small since the tested variants were very rare (MAF < 0.01%) and the cohort size of NASH‐CRN was modest (*N* = 3358), making it relatively underpowered to detect more subtle or less common phenotypic changes. Furthermore, some of the liver related outcomes, such as HCC, were too few to analyse against the rare variant burden. For example, there were 10 cases of incident HCC in our cohort, which made it not possible to replicate a report on *APOB* variants leading to higher risk of HCC [24]. Second, although the total and LDL cholesterol levels between *APOB* carriers and matched non‐carriers were statistically different as expected, the LDL cholesterol in heterozygotes for *LDLR* variants was not markedly higher than those of matched non‐carriers. This may be because of the effect of statin therapy, which was only noted as a yes/no indicator variable without detailed information on the duration, length and dosage information to impute the untreated value. Our adjustment of LDL cholesterol was based on a recent publication in FH patients [16] and although we observed a trend to higher levels of total and LDL cholesterol among heterozygotes for P/LP variants in *LDLR*, these were not statistically significant. Third, we were unable to directly replicate our findings in an independent cohort of patients with MASLD and liver histology information. As an alternative, we screened the UK Biobank for those participants with MRI‐PDFF values instead. Although the direction of the effect of carrying *LDLR* variants trended in opposite directions in NASH‐CRN and the UKBB, we did observe similar trends in related hepatic phenotypes among heterozygotes for *APOB* L/LP variants. Lastly, although this was one of the largest efforts to screen the variants underlying monogenic dyslipidemias in an MASLD cohort, we were not able to verify that most of the variants we curated were associated with the condition. Again, this is because our sample size was modest, indicating that a larger cohort would be needed to identify more individuals who carried such rare variants.

In summary, monogenic disorders of dyslipidemia are rarely present in patients with MASLD and are sometimes associated with worse liver histology and testing for these conditions may be undertaken on a case‐by‐case basis. We found clinically relevant P/LP *APOB* variants that cause hypobetalipoproteinemia and have been associated in other studies with histological progression of liver disease in our cohort of patients with well‐characterised MASLD. Hypobetalipoproteinemia caused by pathogenic variants of *APOB* should be considered in patients with low serum total and LDL cholesterol. Future studies to characterise the roles of less common variants associated with hereditary dyslipidemia would be valuable but likely not feasible given the lack of larger cohorts with detailed histological characterisation of MASLD although this would contribute to a better understanding of their role in progression, risk and outcomes in patients with MASLD.

## Author Contributions

All authors contributed significantly to the development of this manuscript. *Design and concept*: Naga Chalasani, Brent A. Neuschwander‐Tetri, Robert A. Hegele and Tae‐Hwi Schwantes‐An. *Data acquisition*: Marco A. Abreu and Jian Wang. *Analysis and interpretation of data*: Tae‐Hwi Schwantes‐An, Marco A. Abreu, Jian Wang, Xiuqing Guo, Jingyi Tan, Jerry Rotter, Robert A. Hegele and Naga Chalasani. *Manuscript drafting*: Tae‐Hwi Schwantes‐An, Naga Chalasani and Brent A. Neuschwander‐Tetri. *Manuscript critical review*: Robert A. Hegele, Nicholas Davidson, Luca Lotta, Niek Verweij and Jerome I. Rotter. Tae‐Hwi Schwantes‐An and Naga Chalasani had full access to all the data in the study and took responsibility for the integrity of the data and the accuracy of the data analysis.

## Funding

This work was supported by David W. Crabb Endowed Professorship and Terence Kahn Program for Liver Diseases funds to Dr. Naga Chalasani. The NASH‐CRN is supported by the NIDDK (U01DK061713, U01DK061718, U01DK061728, U01DK061731, U01DK061732, U01DK061734, U01DK061737, U01DK0 61 738, U01DK061730 and U24DK061730). No funding was received from the NASH‐CRN for conducting this ancillary study.

## Conflicts of Interest

Dr. Chalasani reports no conflicts for this paper. For full disclosure, he reports consulting agreements with Madrigal, Zydus, Altimmune, Ipsen, Biomed Fusion, GSK, Pfizer, Akero and Boston Pharmaceuticals. He receives research support from Exact Sciences and Boehringer‐Ingelheim. He has equity in Avant Sante, a contract research organisation and Heligenics, a drug discovery start‐up company. Brent A. Neuschwander‐Tetri consults and received grants from Madrigal. He consults for Abbvie, Akero, Aldeyra, Aligos, Arrowhead, Corcept, Galectin, GlaxoSmithKline, Hepion, HistoIndex, Merck, Mirum, Pfizer, Pliant, Sagimet, Senseion and Target RWE. He owns stock options in HepGene and HeptaBio. Linus Schwantes‐An, Xiuqing Guo, Jingyi Tan, Marco Abreu, Nicholas Davidson, Robert Hegele and Jian Wang report no conflicts of interest. Jerome I. Rotter reports equity in Heligenics, a drug discovery start‐up company. Nick Verweij and Luca Lotta are the employees of the Regeneron Genetics Center.

## Supporting information


**Table S1:** Table of the variants included in the manuscript. SPDI, sequence position deletion insertion; Variant, variant name in amino acid changes and nucleotide changes; ClinVarID, ClinVar annotation ID number. Classification: ClinVar classifications, Condition for ACMG classification for the variants in ANGPTL3, APOB, MTTP is Hypobetalipoproteinemia, for the variants in LDLR is Hypercholesterolemia. Criteria Met: ClinGen criteria for recommendation for American College of Medical Genetics and Genomics (ACMG) and Association for Molecular Pathology (AMP). VCEP: ClinGen Variant Curation Expert Panel.
**Table S2:** Table of demographical and clinical details of 24 variant carriers in NASH CRN. ALT, alanine aminotransferase; AST, aspartate aminotransferase; CVD, cardiovascular disease; HbA1c, haemoglobin A1c; HDL, high density lipoprotein; HOMA‐IR, homeostatic model assessment for insulin resistance; LDL, low density lipoprotein; NAS, nonalcoholic steatohepatitis activity score.
**Table S3:** Summary of table of demographics, clinical diagnoses and liver lab values between *APOB* and *LDLR* variant carriers and their matched controls among adult patients subset of NASH CRN. AlkPhos, alkaline phosphatase; ALT, alanine aminotransferase; AST, aspartate aminotransferase; BMI, body mass index; BP, blood pressure; CAD, coronary artery disease; CKD, chronic kidney disease; CVD, cardiovascular disease; FNI, fibrotic NASH index; GGT, gamma‐glutamyl transferase; SD, standard deviation. *APOB* versus matched controls *p*‐value and *LDLR* versus matched controls *p*‐value columns show Wilcoxon rank sum *p*‐values for continuous variables and Fisher's exact *p*‐value for categorical variables.
**Table S4:** Summary of table of demographics, clinical diagnoses and liver lab values between *APOB* and *LDLR* variant carriers and their matched controls among paediatric patients subset of NASH CRN. AlkPhos, alkaline phosphatase; ALT, alanine aminotransferase; AST, aspartate aminotransferase; BMI, body mass index; BP, blood pressure; CAD, coronary artery disease; CKD, chronic kidney disease; CVD, cardiovascular disease; FNI, fibrotic NASH index; GGT, gamma‐glutamyl transferase; SD, standard deviation. *APOB* versus matched controls *p*‐value and *LDLR* versus matched controls *p*‐value columns show Wilcoxon rank sum *p*‐values for continuous variables and Fisher's exact *p*‐value for categorical variables.

## Data Availability

Data presented in this manuscript are available upon request from the corresponding author after an approval from the NASH CRN study review committee.
